# Beclin-1: a therapeutic target at the intersection of autophagy, immunotherapy, and cancer treatment

**DOI:** 10.3389/fimmu.2024.1506426

**Published:** 2024-11-22

**Authors:** Zhumin Cao, Ke Tian, Yincheng Ran, Haonan Zhou, Lei Zhou, Yana Ding, Xiaowei Tang

**Affiliations:** ^1^ Department of Hepatobiliary Surgery, The Seventh People’s Hospital of Chongqing, Chongqing, China; ^2^ Department of Hepatobiliary Surgery, District Traditional Chinese Medicine Hospital, Chongqing, China

**Keywords:** autophagy, Beclin-1, mTOR, autophagy flux, apoptosis and ferroptosis, immunotherapy

## Abstract

The significant identification of Beclin-1’s function in regulating autophagy flow signified a significant progression in our understanding of cellular operations. Beclin-1 acts as a scaffold for forming the PI3KC3 complex, controlling autophagy and cellular trafficking processes in a complicated way. This intricate protein has garnered considerable attention due to its substantial impact on the development of tumors. Strong evidence indicates Beclin-1 plays a critical role in controlling autophagy in various human cancer types and its intricate connection with apoptosis and ferroptosis. The potential of Beclin-1 as a viable target for cancer therapy is highlighted by its associations with key autophagy regulators such as AMPK, mTOR, and ATGs. Beclin-1 controls the growth and dissemination of tumors by autophagy. It also affects how tumors react to therapies such as chemotherapy and radiation therapy. The role of Beclin-1 in autophagy can influence apoptosis, depending on whether it supports cell survival or leads to cell death. Beclin-1 plays a crucial role in ferroptosis by increasing ATG5 levels, which in turn promotes autophagy-triggered ferroptosis. Finally, we analyzed the possible function of Beclin-1 in tumor immunology and drug sensitivity in cancers. In general, Beclin-1 has a significant impact on regulating autophagy, offering various potentials for medical intervention and altering our understanding of cancer biology.

## Graphical Abstract

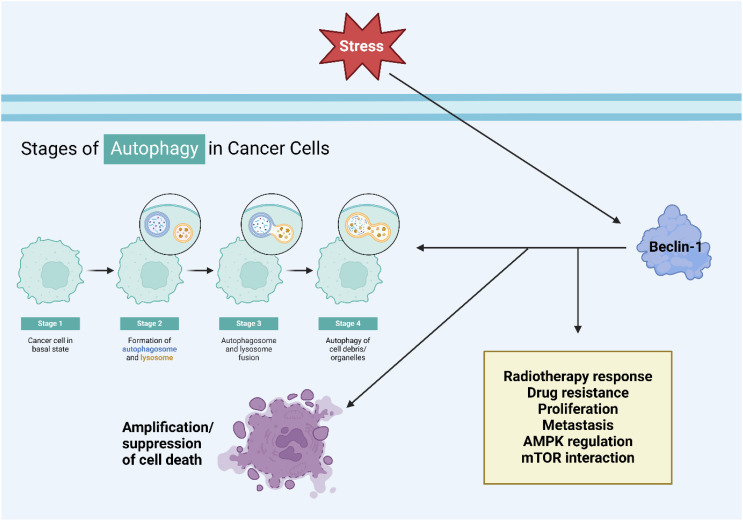

## 1 Introduction

Growing proof has shown that cell death is an essential aspect of cellular existence. Nevertheless, the sophisticated biological studies have showcased the genetically encoded processes that rid the body of unnecessary, permanently damaged, or possibly dangerous cells (1–5). It should be pointed out that regulated cell death (RCD) is not limited to multicellular organisms. Although RCD unquestionably aids in maintaining balance within organisms in normal and abnormal situations, it is also present in simpler forms in single-celled eukaryotes like Dictyostelium discoideum and different yeast species that may gather in colonies, as well as in specific prokaryotes like Escherichia coli. RCD relies on certain molecular machinery, indicating that it can be modified (accelerated or decelerated) through genetic or pharmacological interventions. This is in stark opposition to unintentional cell demise, which is the swift and disastrous death of cells that have been subjected to intense physical, chemical, or mechanical injuries (6–18).

Despite the high level of similarity in the chemical reactions involved, RCD plays a role in two distinct scenarios. RCD can occur spontaneously, serving as a natural part of the body’s growth or tissue renewal processes (7, 19). The term commonly used for these completely natural types of RCD is programmed cell death (PCD). On the other hand, RCD can be activated by changes in the intracellular or extracellular surroundings that are too extreme or extended for the body’s adaptive processes to handle, hindering the restoration of cellular balance. Similarly to adaptive stress responses, stress-induced programmed cell death is an approach used to preserve biological equilibrium (6). Unlike adaptive stress responses, which support cellular and overall homeostasis, programmed cell death (RCD) targets the organism or colony directly, independent of cellular homeostasis (6). This process of maintaining balance in the body involves not just getting rid of harmful or unnecessary cells, but also allows dying cells to release chemicals that alert the organism or colony of potential dangers. DAMPs or alarmins are common terms used to describe molecular patterns associated with damage, serving as warning signals (20–23).

Macroscopic morphological alterations indicate the occurrence of cell demise. In the past, these morphotypes were used to classify cell death into three different types and determine the mechanisms responsible for clearing dead cells and their remnants: (1) apoptosis, characterized by cytoplasmic shrinkage, chromatin condensation, nuclear fragmentation, and plasma membrane blebbing, leading to the formation of apoptotic bodies that are engulfed and degraded by neighboring cells; (2) autophagy, showing cytoplasmic vacuolization and eventual lysosomal degradation after phagocytic uptake; and (3) necrosis, which lacks specific features of apoptosis and autophagy, ending with the removal of cell corpses without phagocytic or lysosomal involvement (24, 25). Nevertheless, despite various limitations and constraints, this morphological classification remains commonly utilized. Starting in 2005, the Nomenclature Committee on Cell Death (NCDD) convened consistently for four reasons: (1) to address problems with a morphology-centered naming of cell death; (2) to accurately delineate key cell death processes using genetic, biochemical, pharmacological, and functional criteria instead of morphology; (3) to distinguish between causes and correlations of cell death; and (4) to set agreed-upon standards for recognizing dead cells with irreversible plasma membrane permeabilization or complete cellular fragmentation as signs of cell death (18, 26–29).

The disruption of cell death processes is a crucial element involved in many diseases, especially cancer. Different pathways of cell death, such as autophagy and apoptosis, interact in intricate ways, impacting the advancement of cancer and how it responds to treatment. This review examines the different functions of autophagy in human cancers, emphasizing its significance as both a protective and detrimental element in tumor biology. Among the numerous regulators of autophagy that have been identified, Beclin-1 is acknowledged as one of the most important. Beclin-1, a vital regulatory protein, is necessary for initiating autophagy and impacts the fine balance between cell survival and death. The present review will comprehensively investigate how Beclin-1 controls autophagy in human cancers, assessing its effects on tumor advancement and drug resistance. Examining Beclin-1’s role reveals cancer cells’ reliance on autophagy for survival. This review explores Beclin-1’s impact on cell death pathways. The connection between these processes is essential in deciding the outcome of cancer cells following medical treatments. Exploring how autophagy affects cancer therapies could lead to new strategies. This review will delve into Beclin-1’s vital role in controlling autophagy, its influence on cancer growth, drug resistance, and interactions with cell death mechanisms. By revealing these connections, we can offer fresh perspectives to enhance the creation of groundbreaking healing strategies for improved treatment of human cancers.

## 2 An overview of cell death

PCD could potentially regulate the equilibrium between cell death and normal cell survival (30). PCD is important in deciding the ultimate destiny of cancer cells when cellular balance is disturbed (31, 32). Noteworthy is the fact that the three main forms of PCD include apoptosis, autophagy, and programmed necrosis. These three forms can be distinguished from each other by their morphological differences. Kerr and colleagues were the first to introduce apoptosis, also referred to as type I PCD. Apoptosis involves specific changes in dying cells, including cell shrinkage, nucleus condensation, membrane blebbing, and detachment from neighboring cells or matrix. The breaking of chromosomal DNA into fragments between nucleosomes, the movement of phosphatidylserine to the outside of the cell membrane, and various internal substrate cleavages due to specific proteolysis are all instances of biochemical changes (33–38). Autophagy, or type II PCD, is a conserved degradation process in various species. It involves the formation of autophagosomes, enclosed by a double membrane, containing macromolecules and organelles for recycling (39–42). Overall, autophagy is crucial in times of famine or stress caused by a lack of growth factors (43), and it is key in preserving cell homeostasis. However, a growing body of evidence indicates that autophagy in cells could experience autolysis, leading to cell death as a response to intense stress. This phenomenon is separate from apoptosis and programmed necrosis (34, 44). Autophagy, acting as “the Janus role,” controls various physiological processes like starvation, differentiation, survival, and cell death (45). Programmed necrosis is a type of programmed cell death that happens alongside apoptosis and autophagy. It is defined by the swelling of cells, dysfunction of organelles, and breakdown of living cells (46–48). Hence, PCD could have a crucial impact on maintaining tissue balance and eliminating unhealthy cells, which is important for cancerous tissues (34).

Typically, apoptosis and autophagy-mediated cell death are considered necessary processes of regulated cell death (49). These processes can lead to organelle malfunction or cell death under conditions of cellular stress. Additionally, they have a crucial role in regulating cancer cell death and specific cancer treatments. Apoptosis plays a crucial role in controlling cell numbers and preserving overall organismal balance (50). Apoptosis is linked to numerous morphological characteristics. These features consist of cell shrinkage, chromatin condensation, membrane blebbing, fragmentation of DNA, and the formation of apoptotic bodies (51, 52). Apoptosis occurs via extrinsic pathway (death receptor activation) and intrinsic pathway (mitochondria control). Ligands like tumor necrosis factor α (TNFα), Fas ligand, and TRAIL trigger extrinsic pathway by binding death receptors on cell surfaces. This activation triggers caspase-8, initiating the final stage of cell death known as apoptosis (53). Proteins in the B-cell lymphoma 2 (Bcl-2) family typically have a function in regulating the intrinsic pathway when there is permanent cellular harm. These proteins control the release of Cytochrome c (Cyt-C) and SMAC/DIABLO, which is a direct IAP-binding protein with low pI, from the mitochondria. The joining of Cyt-C and Apaf-1 proteins triggers caspase 9 activation, causing cancer cell apoptosis (54, 55). Autophagy is an essential phagocytic process for ensuring cell function and balance. Lysosomal fusion can decompose toxic proteins or organelles (56). Autophagy plays two distinct roles in tumor growth, showing that it can either aid or hinder cancer progression, based on the specific tumor subtype and its mutation profile (57). In the pre-cancer stage, inhibiting autophagy leads to the accumulation of ROS and genomic dysfunction. When these two elements are merged, they create pressure on the endoplasmic reticulum (ER) to expand and cause DNA harm, ultimately resulting in tumor development. However, autophagy can provide energy and nutrients to tumors when triggered by a lack of food or oxidative stress (58, 59). Autophagy-related genes, including Beclin-1, light chain 3 (LC3), p62, forkhead box O (FoxO), and Unc-51-like kinase 1 (ULK1), regulate the autophagy process. ULK1 specifically plays a key role in initiating and controlling autophagy, increasing the chances of cancer cell survival (60). Furthermore, autophagy-associated signaling pathways, such as the phosphatidylinositol 3 kinase complex 1 (PI3KC1)-protein kinase B (Akt)-mammalian target of rapamycin complex 1 (mTORC1) pathway, the Ras-Raf-mitogen activated protein kinases (MAPKs) pathway, and the nuclear factor kappa-B (NF-κB) pathway, play a crucial role in fighting against tumor advancement and spread of metastasis. Autophagy and apoptosis are the main mechanisms that control cell destiny and uphold cellular balance within the cell. Alternatively, there is a distinct relationship between apoptosis and autophagy that can result in cell death via methods that are separate or supportive (61). The controlled management of apoptosis and autophagy has proven the therapeutic effectiveness of small-molecule compounds in the development of cancer treatments (62). For instance, studies have demonstrated that Ampelopsin (Amp) triggers apoptosis and autophagy-related cell death in glioma cells by stimulating ROS production and activating c-Jun N-terminal kinase (JNK) (63). Galectin-1, a member of the galactose lectins family, carries out a range of biological functions. This protein, present in many tumor structures, regulates the proliferation, migration, and growth of tumor cells (64). **Table 1** outlines the pathways of cell death involved in cancer.

**Table 1:** Cell death pathways in cancer.

Cell death pathway	Tumor	Summary	Ref
Immunogenic cell death	Lung cancer	ERO1A ablation accelerates endoplasmic reticulum stress and mediates Immunogenic cell death to potentiate immunotherapy	(65)
Immunogenic cell death	Colorectal cancer	Acceleration of immunogenic cell death by aspirin in cancer immunotherapy	(66)
Immunogenic cell death	Hepatocellular carcinoma	Lenvatinib promotes Immunogenic cell death and mediates toll-like receptor-3/4 ligands	(67)
Immunogenic cell death	Lung cancer	Afzelin suppresses NQO2 to mediate cell death	(68)
Immunogenic cell death	Lung cancer	Marsdenia tenacissima extract can enhance ER stress-related immunogenic cell death through AXL downregulation	(69)
Apoptosis Ferroptosis	Colorectal cancer	Silencing CAPG can enhance apoptosis and ferroptosis through p53 downregulation	(70)
Apoptosis	Colon cancer	Metformin and O-GlcNAcylation inhibitor can synergistically mediate apoptosis	(71)
Apoptosis	Pancreatic cancer	SDR16C5 can suppress growth and metastasis, while it induces apoptosis	(72)
Apoptosis	Esophageal cancer	Propofol accelerates apoptosis and diminishes proliferation through caspase-3/7 upregulation	(73)
Apoptosis Autophagy	Thyroid cancer	Silencing KAT5/KIF11 can enhance apoptosis and autophagy	(74)
Apoptosis	Liver cancer	Blue light irradiation increases ROS levels and upregulates Bax and Bad to mediate apoptosis	(75)
Ferroptosis	Oral cancer	FTO downregulates ACSL3 and GPX4 to mediate ferroptosis	(76)
Ferroptosis	Colon cancer	PR-619 as deubiquitinase inhibitor stimulates ferroptosis to boost immunotherapy	(77)
Ferroptosis	Hepatocellular carcinoma	Fe^3+^-binding transferrin nanovesicles can deliver sorafenib and promote lipid peroxides to mediate ferroptosis	(78)
Autophagy	Thyroid cancer	miR-363-3p suppresses autophagy and cancer malignancy through NF-κB downregulation	(79)
Autophagy	Breast cancer	Bruceine A controls PI3K/AKT axis to mediate autophagy	(80)
Autophagy	Lung cancer	Resveratrol increases paclitaxel sensitivity by autophagy induction through regulation of PINK1/Parkin axis	(81)
Autophagy	Endometrial cancer	ICG-001 stimulates autophagy and impairs cell cycle progression	(82)
Autophagy	Breast cancer	Lentinus edodes release β-glucan that suppresses M2 polarization of macrophages via enhancing autophagy cell death	(83)

Researchers developed chitosan nanoparticles that respond to dual pH to enhance drug delivery and accumulation in MDR breast cancer cells, named DCCA/DOX-NPs. These nanoparticles exhibited increased cellular absorption, heightened toxicity, and enhanced antitumor effectiveness in living organisms by overcoming resistance mechanisms (84). In hepatocellular carcinoma, sorafenib and its derivative SC-59 both trigger autophagy through SHP-1-STAT3-Mcl-1-Beclin 1 pathway, with SC-59 showing greater efficacy in decreasing cancer cell viability and tumor growth (85). The use of Adenovirus vector to express XAF1 in gastric cancer cells promotes autophagy through increasing Beclin-1 levels and inhibiting the Akt/p70S6K pathway, leading to enhanced apoptosis simultaneously (86). Rhus coriaria extract (RCE) exhibits notable effectiveness in fighting colorectal cancer cells by diminishing cell viability and colony formation, triggering Beclin-1-independent autophagy and caspase-7-dependent apoptosis, impacting the AKT/mTOR pathway, and influencing crucial proteins related to proteasome-mediated breakdown (87). Tanshinone IIA (TAN) slows the growth of oral squamous cell carcinoma (OSCC) by inducing cell death and self-cleaning mechanisms through specific pathways, while also decreasing the activity of other pathways, with its effectiveness being linked to Beclin-1 levels (88). Cisplatin enhances autophagy and apoptosis in A549 human lung cancer cells by increasing Beclin 1 and Atg5, while blocking these autophagy-related proteins worsens apoptotic cell death (89). Autophagy may promote programmed cell death in cancer cells exposed to cobalt chloride (CoCl2)-induced hypoxia through an ATG5-dependent mechanism, which includes crosstalk with endoplasmic reticulum stress and mitochondrial pathways, and identifies two separate autophagy-related routes that could aid in the creation of new anti-cancer treatments (90). Thus, Beclin-1 controls cell death in human cancers (91–93).

## 3 Autophagy flux

Autophagy, frequently referred to as the self-recycling process, involves complex lysosome-dependent pathways in eukaryotic cells to control protein, lipid, and organelle levels. Autophagy originates from the Greek terms “auto” and “phagein,” denoting “self-consumption.” When the body experiences a lack of nutrition or energy, it initiates autophagy binge to compensate. Autophagy can also be triggered by various cellular stressors, including the buildup of harmful protein clusters that are specifically broken down through autophagic processes. In the same way, lysosomes can engulf cellular organelles like LDs through autophagy processes to create metabolites. Hence, autophagy serves as an important control mechanism for a range of cellular and tissue functions, such as growth, coping with stress, immune reactions, and metabolic processes. Furthermore, there are three forms of autophagy: macroautophagy/autophagy, microautophagy, and chaperone-mediated autophagy (CMA) (94, 95).

Autophagy dysfunction is associated with illnesses such as neurodegeneration, macular degeneration, Crohn’s disease, and cancer. It plays crucial roles in controlling cellular growth, metastasis, and treatment resistance in cancer. As a result, autophagy has garnered significant attention in cancer treatment, with researchers focusing on regulating it using nanoparticles and anti-cancer medications (96–100). Additionally, aging is linked to malfunctions in the autophagy process. Autophagy is divided into three types: (1) Microautophagy involves the direct ingestion of cellular material by lysosomes. (2) Chaperones selectively recognize substrate proteins, then unfold and translocate them to lysosomes through a lysosomal receptor, and (3) Macroautophagy involves non-selectively enclosing bulk cytoplasm in double-membraned vesicles, which later fuse with lysosomes for degradation. Additional information on autophagy can be found in several scientific reviews (101–104). Many eukaryotic organisms have over 90% of their cells participating in basic macroautophagy processes. Macroautophagy starts with a precursor structure, known as the phagophore, that eventually transforms into an autophagosome. The recently created autophagosome combines with lysosomes, which then expose the internal cytosolic material to enzymes that degrade it in an acidic setting. Autophagosomes gather the lipids they need for formation from different organelles within the cell including the Golgi complex, mitochondria, recycling endosomes, the ER, and even the plasma membrane. Proteins called autophagy-related (ATG) proteins supply these lipids in vesicular form (105–109). Yoshinori Ohsumi, Daniel Klionsky, and Michael Thumm were the first to discover and characterize ATG genes (109–111). Activation of the ULK1/Atg1 kinase complex is necessary to initiate the process of autophagosome formation in autophagy. The ULK1 kinase complex primarily incorporates signals from two kinases: mTORC1 and PRKA/AMPK, both of which are upstream molecular signals. These complexes are vital in controlling the initiation of the autophagy process (112–114). Another part of creating a phagophore from scratch includes its growth in particular ER subdomains referred to as omegasomes. The omegasomes need the ULK1 kinase complex to be activated in order to form (115). The second group acts as a nursery for the growth of autophagosome (116).

The ULK1 complex phosphorylates and activates a different protein kinase complex, called the class III phosphatidylinositol 3-kinase (PtdIns3K) complex (BECLIN1/Vps30/Atg6-PIK3R4/Vps15-ATG14-PIK3C3/Vps34-AMBRA1). When Beclin-1/Vps30/Atg6 combines with PIK3R4/VPS15 and ATG14, PIK3C3/VPS34 is triggered to produce PtdIns3P. Proteins such as DFCP1 (zinc finger FYVE-type containing 1) (115) and phosphoinositide interacting (WIPI) are connected through binding proteins to participate in cellular processes (117). WIPI2 and WIPI1 collaborate to bring in the ATG12-ATG5 complex, together with ATG16L1 (118). ATG16 combines with the ATG12-ATG5 complex to initiate the formation of phagophores (119). The elongation complex (WDR45B/WIPI3 and WDR45/WIPI4) and WDR45, specifically interact with ATG2 (118, 120). The ATG12-ATG5-ATG16L1 conjugate acts as an E3-like ligase (109), to attach MAP1LC3 from the LC3 protein family to phosphatidylethanolamine (119). The LC3 protein family is made up of seven members, with four in the MAP1LC3 group (LC3A, LC3B, LC3C, and LC3D) and the other three in the GABA Type A receptor-associated protein groups (GABARAP, GABARAPL1, and GABARAPL2), representing GABA Type A receptor-associated protein and GABA type A receptor associated protein like 1 and 2, respectively. In terms of function, LC3 proteins play a role in selecting cargo, elongating, and closing phagophores (119, 120), resulting in the development of fully mature autophagosomes that merge with lysosomes to form autolysosomes (121).

## 4 Evolution of autophagy genes

The origins of all three domains of life - bacteria, archaea (prokaryotes), and eukaryotes - can be traced back to the last eukaryotic common ancestor (LECA). Thaumarchaeota is one of the primary branches of archaea, followed by Asgard, and then Crenarchaeota, Korarchaeota, and Aigarchaeota (TACK) (122). Prokaryotes lack the autophagy pathway found in eukaryotic cells. This system relies on the existence of intracellular membrane compartments. The diverse functions seen within eukaryotic lineages are probably due to the presence of most, if not all, core autophagy related (ATG) proteins in the Last Eukaryotic Common Ancestor (LECA). Not every subgroup participates in autophagy, but it is likely that early on in the LECA era, two ATG protein families - Atg1/ULK and PROPPINs - which interact with polyphosphoinositides, had already branched out into various subgroups. Plants and vertebrates experienced numerous duplication events. In the usual ATG conjugation systems, ATG12 is bonded to ATG5 through a covalent attachment. Nevertheless, essential components required for this conjugation are absent in Toxoplasma and Plasmodium, which are part of the Alveolata within the SAR supergroup of Stramenopiles, Alveolata, and Rhizaria, as well as in Komagataella, a yeast genus. Specifically, the E2-like enzyme ATG10 and the C-terminal glycine of ATG12 are not present. Therefore, these organisms depend on noncovalent connections involving ATG12 and ATG5 instead (123, 124). The noncovalent form is considered adaptable because it does not depend on enzymes or ATP. Eukaryotic organisms might have experienced as many as sixteen transitions from covalent to noncovalent bonds.

*Saccharomyces cerevisiae*, frequently utilized in studies on budding yeast, is commonly acknowledged as the gold standard in autophagy investigations. This reputation is because of its crucial involvement in breakthroughs like the discovery of the ATG gene. Research indicates autophagy in S. cerevisiae is different from other species.

In different kinds of organisms, Atg1 complex consists of Atg29 and Atg31 in addition to Atg17. Nevertheless, the S. cerevisiae Atg1 complex does not have ATG101 like other species (125), preventing the stabilization of ATG13 and the formation of a complex. It is not clear if the loss of ATG101 is related to the acquisition of Atg29 and Atg31. S. cerevisiae does not have VMP1, a crucial protein for autophagy in various other organisms such as metazoa, Dictyostelium, and potentially green algae. VMP1 is located in the ER and is situated after the hisT gene in the Escherichia coli DNA gene A superfamily. Crucially, S. cerevisiae employs a distinctive biosynthetic mechanism called the cytoplasm-to-vacuole targeting (Cvt) pathway for delivering vacuolar hydrolases to the vacuole. Furthermore, Schizosaccharomyces pombe also contains an alternative metabolic pathway called the Nbr1-mediated vacuolar targeting (NVT) pathway.

Furthermore, proteins associated with specific autophagy have been discovered to increase in gene families. NBR1 (Atg19 in S. cerevisiae) is found in various eukaryotic organisms, and SQSTM1 likely evolved from duplication of NBR1 and loss of NBR1 domains. The presence of OPTN and CALCOCO families in the majority of metazoan species indicates that they underwent expansion in vertebrate evolutionary lineages. While most ATG proteins are found only in eukaryotic organisms, certain ones may have originated in prokaryotes. Many functional complexes in the autophagy pathway have proteins in common with distant prokaryote counterparts. Various instances of this phenomenon are demonstrated, including the DedA superfamily proteins (TMEM41B and VMP1), the Hop1, Rev7 and Mad2 (HORMA)-domain-containing proteins (ATG13 and ATG101), transmembrane segment of ATG9, chorein-N domain at the N termini of lipid transfer proteins (ATG2), and ubiquitin-like ATG conjugation systems. This implies that autophagy developed partially through the utilization of genes already present. Prokaryotes are the ancestors of the endosomal sorting complex required for transport (ESCRT) proteins, crucial for micro- and macroautophagy. Even though ESCRT-I, -II, and -III function sequentially in eukaryotic cells during membrane fission, bacterial and archaeal proteins like PspA/Vipp1 and CdvB appeared earlier in evolution. Although the Asgard archaea group is believed to be the initial habitat of the ESCRT-I and -II proteins, they were introduced at a later time. Therefore, it is believed that eukaryotic organisms evolved from the Asgard group, which had a complete ESCRT system (although lacking ESCRT-0, found only in Opisthokonta) (123, 126–129).

## 5 Autophagy in cancer drug resistance

Inhibiting autophagy may enhance tumor cells’ responsiveness to regular medications or combat the resistance they have built up against chemotherapy (130–132). In the upcoming section, we will explore the latest and most relevant findings on combining various anticancer drugs with autophagy inhibitors and activators. Combining CQ and HCQ with medicines like 5-fluorouracil (133), cisplatin (134), and temozolomide (135, 136), enhances their cytotoxicity. Additionally, the use of both CQ and trastuzumab together was able to block tumor growth by over 90% in a HER2-positive breast cancer tumor xenograft that had total resistance to trastuzumab (137). Furthermore, different autophagy blockers have demonstrated positive outcomes, such as verteporfin’s improvement of gemcitabine’s effectiveness in an *in vitro* model of pancreatic cancer (138). SBI-0206965 was able to overcome resistance to cisplatin in NSCLC cells (139) as well as resistance to cabozantinib in metastatic colorectal cancer (140). The combination of celecoxib, a specific inhibitor of cyclooxygenase-2, with CQ and SAR405 led to an enhancement in cell death (141). Furthermore, research demonstrated that 3MA increased the level of cell demise caused by bortezomib in glioblastoma cells (142). Furthermore, the use of CQ analog lys05 with the second generation tyrosine kinase inhibitor, nilotinib, has shown to have an extra effect on reducing the amount of leukemia stem cells in mouse models of chronic myelogenous leukemia (143). By inhibiting autophagy, UAMC-2526 can improve the effectiveness of oxaliplatin in a colorectal cancer mouse model. In addition, UAMC-2526 treatment led to a more differentiated phenotype in tumors (144). Research has investigated using both autophagy activators and inhibitors together; for instance, CQ and HCQ have been shown to boost the impact of mTOR inhibitors like temsirolimus (145) or everolimus (146), in colorectal cancer (147), melanoma (145), and neuroendocrine neoplasms (146). This indicates that blocking autophagy can be utilized to address resistance to mTOR inhibitors. In addition, research has shown that CQ can improve the cancer-fighting abilities of vorinostat (148). Moreover, the synergistic effects of combining everolimus with SAR405 have been shown in studies (149, 150). In light of these observations, it can be inferred that suppressing autophagy proves to be a more efficient approach to treatment. This happens due to the fact that autophagy has a protective function in the models being studied. Autophagy inducers, however, can also help overcome chemoresistance, although to a lesser extent. Studies have demonstrated that Temsirolimus can enhance the efficacy of gemcitabine and cisplatin in bladder cancer cell lines (151). Furthermore, studies have demonstrated that it can decrease the susceptibility of colon cancer cells to cetuximab (152). In the same way, curcumin has been found to enhance the effectiveness of gefitinib in primary gefitinib-resistant small-cell lung cancer cells through autophagy-dependent synergism (153). However, studies conducted before clinical trials have revealed that controlling autophagy can lead to conflicting outcomes under specific circumstances. Lab research shows mTOR + ULK1 inhibitor combo induces A549 cell apoptosis (154, 155). SBI-0206965 sensitizes neuroblastoma cells to TRAIL, not mTOR inhibitors. This implies that autophagy does not have a protective function within this model (155). At this point, it is essential to utilize molecular markers that can anticipate how tumors will react to autophagy modulators. One illustration of such an indicator is the BRAF V600E mutation, linked to protective autophagy (156).

5-FU, antimetabolite chemo used long term for solid tumors like head/neck, breast, GI, and pancreatic cancer (157, 158). In S phase, halts thymidylate synthetase, decreasing thymidylate for DNA replication, affecting cell cycle progression (159). Nevertheless, the effectiveness of 5-FU is limited by the promotion of protective autophagy as an unintended consequence in different cancer types through multiple pathways: (1) augmentation of Beclin-1 expression, aiding in the transformation of LC3I to LC3II (160); (2) JNK-triggered protective autophagy and activation of Bcl-2 phosphorylation, resulting in resilience to 5-FU; and (3) increased autophagy flow when 5-FU is present (161). Despite its purpose of damaging DNA in the guanine residue, TMZ, an alkylating medication utilized for treating glioma (162), often shows restricted effectiveness. This is partly because it triggers protective autophagy, which may play a role in building resistance (163, 164). Following temozolomide treatment, various mechanisms have been demonstrated to trigger protective autophagy. Some of these mechanisms involve ATM increasing the AMPK-ULK1 pathway (165), generating ROS, activating the signal-regulated kinase (ERK) pathway (166), and creating mitochondrial and ER stress (167). Cisplatin-based chemotherapeutic regimens are standard treatments for solid tumors due to their DNA damage and mitochondria apoptosis effects, although drug resistance can limit their effectiveness (168, 169).

Autophagy plays a significant role in various pathways of cisplatin-induced chemoresistance, specifically in ovarian cancer, where it is triggered by the activation of the ERK pathway (170), and the upregulation of Beclin-1 (171). Cisplatin induces protective autophagy in esophageal cancer by increasing Beclin-1 levels, converting LC3-I to LC3-II (172), and upregulating ATG7 expression (173). Consequently, inhibiting autophagy along with cisplatin treatment results in increased cell death in esophageal cancer (174).

## 6 Major regulators of autophagy machinery

### 6.1 Beclin-1

Beclin-1 is a new protein containing only the BH3 domain of Bcl-2 (175). Unlike other animals lacking autophagy genes, which die in early embryo development (before E7.5) because of issues with proamniotic canal closure, Beclin-1 null mice have a significantly more severe embryonic phenotype (176). Beclin-1 located in cytoplasm, ER, mitochondria, and perinuclear membranes. Found in various human and mouse tissues. Also seen in different regions of human colon cancer tissue via immunohistochemistry (177). Beclin-1 is comprised of three specific structural regions: a BH3 domain at the N-terminal (amino acids 114-123), a coiled-coil domain (CCD) (amino acids 144-269), and an evolutionarily conserved domain (ECD) (amino acids 244-337). ECD domain important for Beclin-1 to regulate autophagy, prevent cancer.

**Figure 1:**
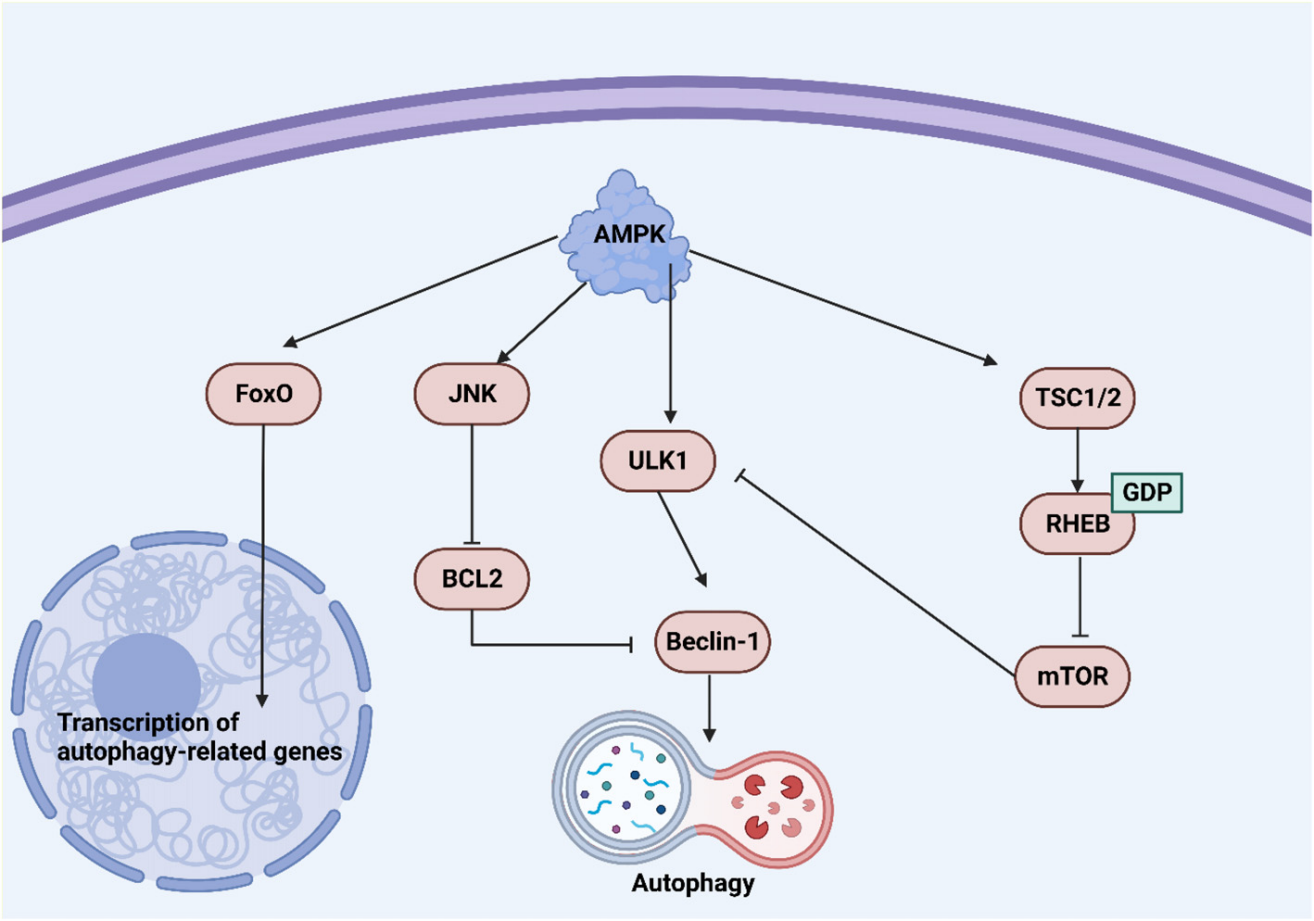
An overview of Beclin-1 in autophagy. Upregulation of Beclin-1 can induce autophagy. AMPK upregulates ULK1 to increase Beclin-1 levels for autophagy induction. On the other hand, an increase in mTOR levels can suppress ULK1 to reduce Beclin-1 levels in autophagy suppression.

The short leucine-rich amino acid sequence makes the nuclear export signal (NES) of Beclin-1 effective. The Beclin-1 NES mutation hinders its capacity to induce autophagy during food scarcity and to inhibit cancer progression. Twelve Bcl-2 family members, known for their ability to prevent cell death, bind to the BH3 domain of Beclin-1. Activators such as activating molecule in Beclin-1-regulated autophagy (Ambra1), UV radiation resistance-associated gene (UVRAG), and Atg14L interact with the CCD, while both the ECD and CCD are connected to PI3KC3/Vps34 (**Figure 1**, **Table 2**) (191, 192). Beclin-1 not only controls autophagy, but also regulates mitophagy, a specific form of autophagy (193).

**Table 2:** Beclin-1-mediated regulation of cancer progression.

Tumor type	Molecular aspects	Remark	Ref
Urinary bladder cancer	p53/PCDH17/Beclin-1	The downregulation of p53 and PCDH17 can mediate muscle-invasive bladder cancer Beclin-1 determines the T stage The patients with p53 mutation, PCDH17 downregulation and Beclin-1 poor expression have poor survival rate	(178)
Gastric cancer	Beclin-1	Beclin-1 stimulates apoptosis and decreases metastasis	(179)
Ewing sarcoma	Beclin-1	Silencing Beclin-1 downregulates MMP-9 to reduce metastasis	(180)
Non-small cell lung cancer	Beclin-1	Beclin-1 shows low expression, while Bcl-2 is upregulated in lung tumor	(181)
Esophageal cancer	Beclin-1	Upregulation of Beclin-1 enhances occurrence and development of cancer	(182)
Colorectal cancer	Beclin-1	Upregulation of Beclin-1 triggers desirable prognosis	(183)
Cholangiocarcinoma	Beclin-1 ARID1A	Upregulation of ARID1A and Beclin-1 mediate undesirable survival and tumor relapse	(184)
Thyroid cancer	Beclin-1	Beclin-1 upregulation increases potential of proteasome inhibitors in cancer therapy	(185)
Breast cancer	Akt Beclin-1	Selenium suppresses proliferation through decreasing levels of Akt and Beclin-1	(186)
Primary duodenal adenocarcinoma	Beclin-1	Upregulation of Beclin-1 enhances drug sensitivity and mediates desirable outcome	(187)
Colorectal cancer	Beclin-1	Beclin-1 expression is related to the distant metastasis	(188)
Breast cancer	miR-124-3p Beclin-1	miR-124-3p downregulates Beclin-1 and LC3I in the cancer therapy	(189)
Lung cancer	Beclin-1	Apoptosis induction and metastasis inhibition by Beclin-1	(190)

Beclin-1 is crucial for maintaining cellular balance and controlling autophagy, acting as a key center that combines different cellular cues. Its dual role involves promoting autophagy cell death and also inhibiting apoptosis by interacting with the Bcl-2 protein family. Maintaining a delicate balance between these processes is essential for how cells respond to stress and keep tissues healthy. Beclin-1 plays a crucial role in autophagy and is involved in various cellular processes like inflammation and immune responses. Its significance extends beyond autophagy regulation and dysfunction is linked to cancer. Targeting Beclin-1 for treatments may offer new approaches in cancer therapy, making it a potential biomarker and therapeutic target.

### 6.2 mTOR

**Figure 2:**
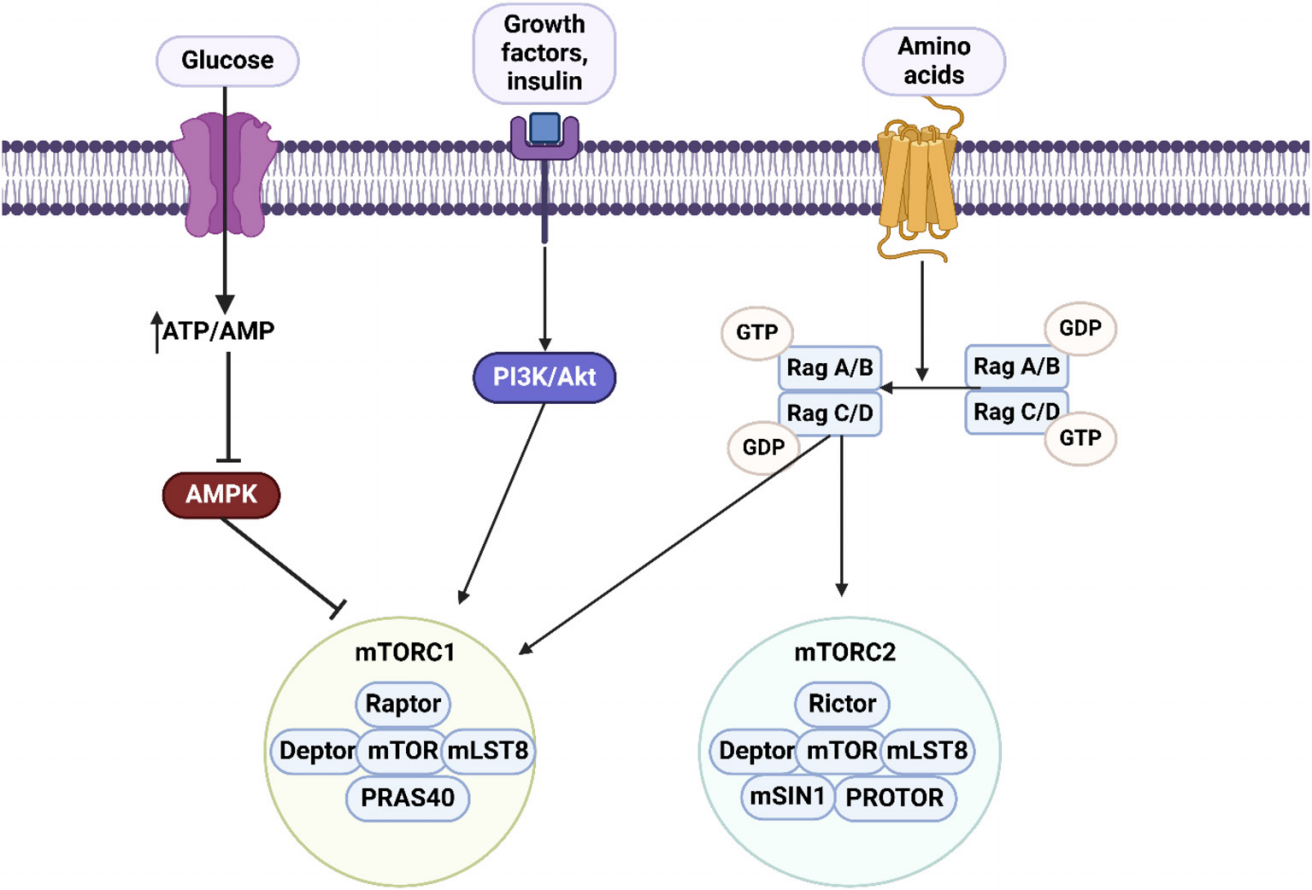
An overview of mTOR axis. There are two complexes including mTORC1 comprised of Raptor, Deptor, mTOR, mLST8 and PRAS40, and mTORC2 comprised of Rictor, Deptor, mTOR, mLST8, mSIN1 and Protor. The upstream regulator of mTORC1 is AMPK which suppresses the mTORC1 axis. Lack of glucose and an increase in ATP/AMP levels can regulate the AMPK/mTORC1 axis. Changes in amino acid levels can affect both mTORC1 and mTORC2 levels. Moreover, PI3K/Akt axis is considered as the upstream regulator of mTORC1. Therefore, most of the molecular pathways are related to mTORC1.

Cell growth and metabolism are influenced by hormonal, nutrient, and energy signals via signaling pathways and transcription factors. mTOR, a crucial kinase, regulates cellular metabolism and responds to environmental cues, with its overactivity associated with diseases. mTOR interacts with multiple proteins to create two complex entities known as mTOR complex 1 (mTORC1) and mTORC2 (194). A fundamental list of individuals found in both mTORC1 and mTORC2 consists of the Tti1/Tel2 complex, mLST8, and DEPTOR, all functioning in both groups (195–198). RICTOR, mSin1, and PROCTOR1/2 are unique to mTORC2, whereas RAPTOR and PRAS40 are dedicated solely to mTORC1 (199–209). Two kinase complexes can activate signal cascades and control cellular activities independently based on various substrate preferences. mTOR creates two distinct signaling complexes called mTORC1 and mTORC2. One function of mTORC1 is to integrate signals from growth factors and nutrients to promote increased anabolic metabolism, including elevated protein and lipid synthesis, and inhibition of autophagy or lysosome production. The TSC complex prevents mTORC1 activation by stimulating Rheb GTPase, a requirement for mTORC1 activity. The compound is blocked by signaling pathways triggered by growth factors like PI3K/AKT or Ras/Erc, or stimulated by cellular stressors that involve mTORC1. During stressful situations like low energy or oxygen levels, AMPK activates and also inhibits the TSC complex by converting ATP to AMP. This inhibition also includes AMPK’s phosphorylation of RAPTOR, which directly impacts mTORC1. Moreover, Rag GTPases move mTORC1 to the lysosomal surface upon amino acid stimulation, where Rheb GTPase subsequently triggers its activation. On the other hand, mTORC2 is activated by growth factor/RTK/PI3K signaling and has important functions in cell metabolism, the cytoskeleton, and survival through AGC family kinases. Activation of mTORC1 has the ability to block IRS and enhance GRB10 functionality, leading to the suppression of RTK/PI3K/AKT signaling. An mTORC1 effector, S6K, additionally suppresses IRS and indirectly impacts mTORC2 via inhibitory phosphorylation (**Figure 2**) (210).

### 6.3 AMPK

AMPK is believed to be made up of three parts - α, β, and γ subunits - with slight structural differences seen in various species (211). The α subunit of AMPK has a Ser/Thr kinase domain at the start of the protein. Phosphorylation at Thr-172 is crucial for regulating its activity in humans. LKB1, TAK1, and CaMKKβ are upstream kinases targeting Thr-172. Circac1 also plays a role in controlling AMPK function (212). Thr-172. Circac1 is also involved in controlling AMPK function (212). Moreover, AMPK also includes multiple allosteric activation sites. These consist of the self-inhibiting domain (AID) on the α subunit, as well as domains for interacting with the β and γ subunits. The main functions of the β-regulatory subunit are found in two key areas: the carbohydrate binding region (CBM) which helps with energy supply, and the end area which links to the α and γ subunits. The γ regulatory subunit contains multiple cystathionine-β-synthase (CBS) tandem repeats that make up the Bateman domain, which is essential for detecting energy levels and interacting with ATP and AMP (213). Nutrient depletion will result in elevated AMP/ATP ratio and lowered ATP levels. It was previously believed that activation of AMPK γ subunit required extremely high levels of 5′-AMP when AMP was present in millimolar concentrations (214). AMPK Thr-172 can be targeted by a very high concentration of AMP through calcium ion-dependent CaMKKβ and AMP through the AMP-dependent LKB-1. Before, it was thought that AMPK only controlled bioenergetic metabolism, but now it is understood that it also protects the nervous system, lengthens telomeres, triggers the tumor suppressor p53, and improves mitophagy and synthesis. It has been experimentally demonstrated that boosting the AMPK signaling pathway in the body-wall muscles of nematodes can extend their average lifespan. Moreover, the longevity of the nematode is prolonged by neuronal TORC1 through the reduction of mitochondrial activity by activating AMPK (215). Alpha-ketoglutarate (AKG) prolongs the lifespan of fruit flies by blocking the mTOR pathway and boosting the AMPK signaling pathway when glucose is transformed into blood sugar in periods of high energy, ultimately raising insulin levels. Insulin indicates a surplus of nutrients, causing cells to absorb and use more resources by attaching to receptors on the cell surface and initiating the PI3K-AKT pathway through IRS1. The AKT-TSC1/2-RheB-mTORC1 pathway is known to be the route through which insulin inhibits AMPK activity and stimulates mTORC1 (216).

### 6.4 ULK1

**Figure 3:**
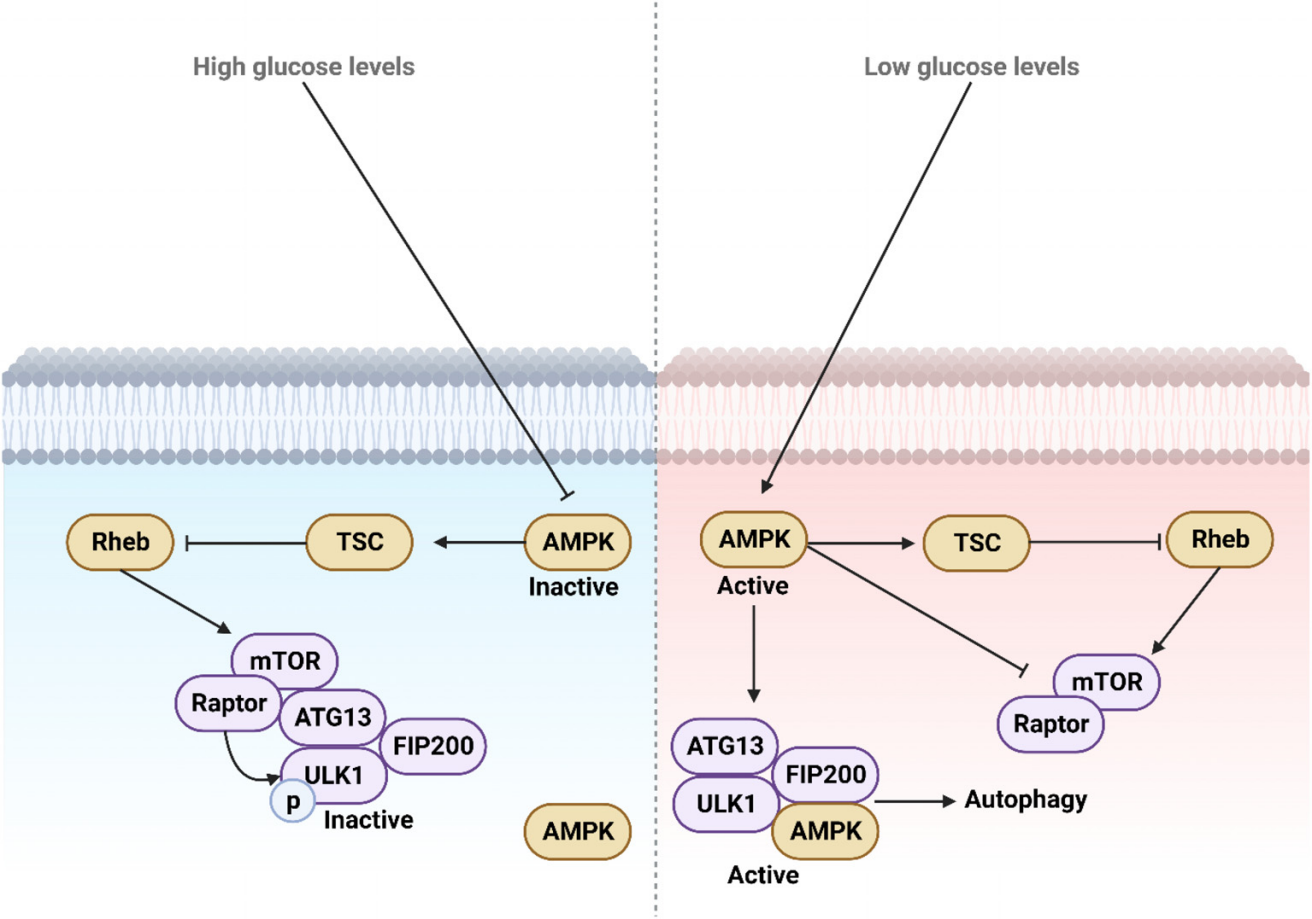
The function and regulation of ULK1 during low and high glucose levels. High glucose levels deactivate AMPK by suppressing its activity, whereas low glucose levels activate and upregulate AMPK, which in turn mediates autophagy. This process provides necessary energy through the degradation of macromolecules and aged organelles.

Only one autophagy-linked kinase has been identified in S. cerevisiae, which is known as Atg1 and is a conserved serine/threonine kinase (217, 218). Atg1 loss leads to early termination of yeast autophagy (218). In mammals, Atg1 is similar to ULK1 and ULK2, which are kinases that resemble uncoordinated-51. ULK1 and ULK2 work together; the absence of ULK1 results in a slight phenotype in mice (219). In mammals, Papinski and Kraft (2016) (220), discovered that the start of autophagy is triggered by ULK1 combining with FIP200, ATG13, and ATG101. ULK1 activity is regulated by AMPK and MTORC1 as energy and nutrient sensors. In environments with abundant nutrients, MTORC1 suppresses autophagy, but fasting or rapalog treatment inhibits MTORC1, causing an increase in ULK1 kinase activity in mammalian cells (221). Studies indicate that MTORC1 might regulate the phosphorylation of two proteins in the initiation complex, ULK1 and ATG13 (112). The discovery was made that MTORC1 phosphorylation on Ser757 can inhibit the activity of ULK1 and interfere with its interaction with AMPK (222). AMPK phosphorylation initiates autophagy by activating ULK1, in contrast to MTORC1 phosphorylation. During amino acid deprivation, AMPK adds phosphate groups to ULK1, assisting in the degradation of mitochondria via mitophagy (223). AMPK phosphorylates ULK1 at Ser555, regulating hypoxia-triggered autophagic breakdown of mitochondri (224). ULK1 phosphorylates autophagy regulators. ULK1 phosphorylates its binding partners ATG13, FIP200, and ATG101. The PI3KC3 complex 1, which is specific to autophagy, consists of Beclin-1, Vps34/PIK3C3, and Ambra, all phosphorylated by ULK1 (220). Phosphorylation of FUNDC1 by ULK1 is crucial for promoting the proper association of the mitophagy adapter LC3 with FUNDC1, which helps advance mitophagy (225). Additionally, ULK1 helps maintain autophagy flow by triggering a feedback inhibition of the MTORC1 complex via phosphorylating Raptor, ultimately leading to its inhibition (226). AMPK phosphorylation accompanies ULK1-regulated autophagy, leading to the formation of a negative feedback loop (**Figure 3**) (227).

### 6.5 Autophagy-related genes

**Figure 4:**
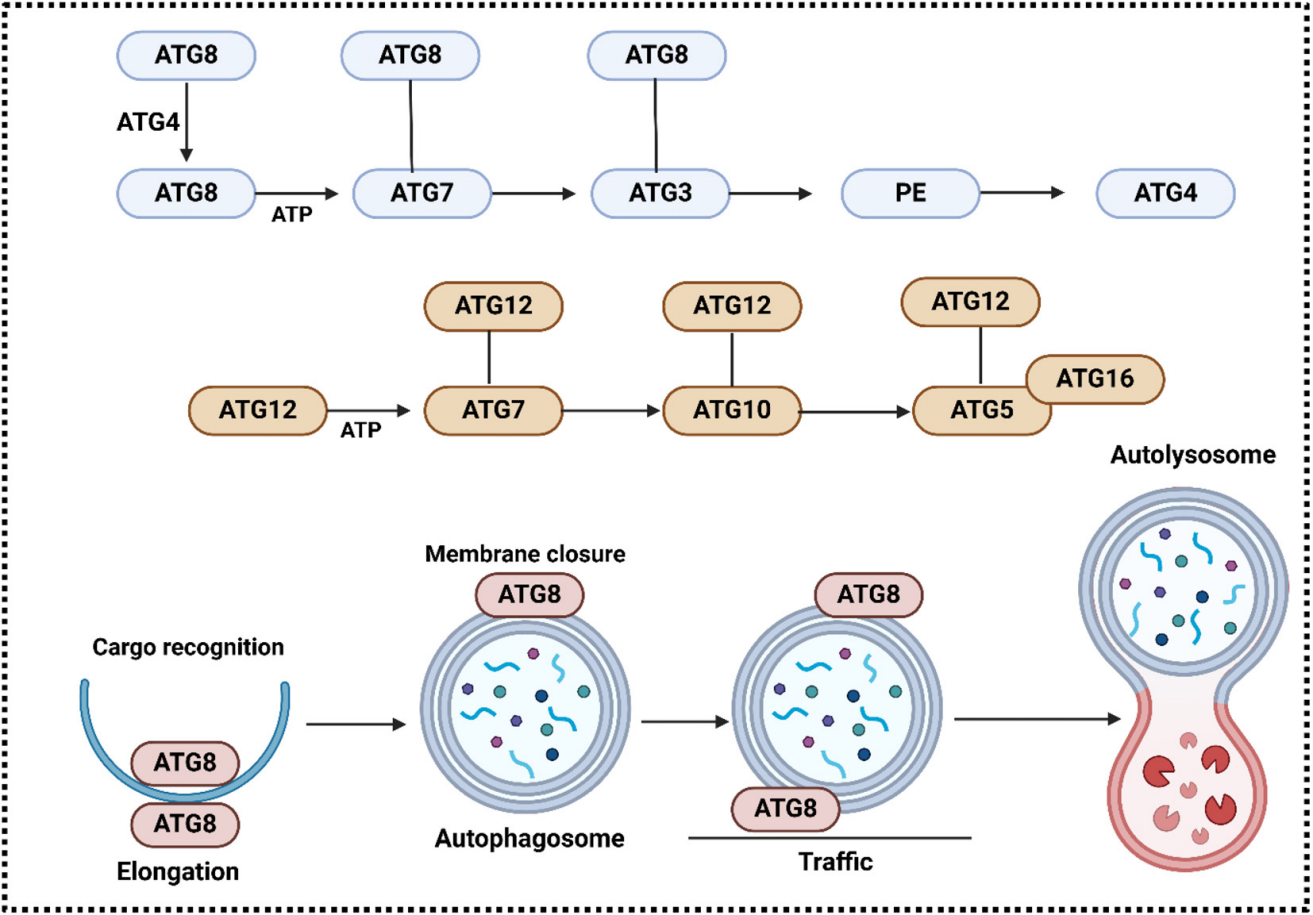
The involvement of ATG system in the regulation of autophagy. ATG7 (E1-like) activates ATG12 in an ATP-dependent way, and a high-energy thioester bond couples its C-terminal glycine to the cysteine of ATG7 active site. Another thioester intermediate is formed when ATG12 is transferred to ATG10 (which is E2-like). An isopeptide bond binds ATG12 to the acceptor lysine in ATG5. Connected to ATG16(L) is ATG5. The ATG12-ATG5-ATG16 complex is formed when ATG16(L) forms a dimer. Following proteolytic processing by ATG4 enzymes, the ATG8 system proceeds to activate the ATG8 protein(s), which in turn produce a thioester with the E2-like enzyme ATG3. In the end, phosphatidylethanolamine (PE) is bound to ATG8. autophagy membranes contain the conjugated ATG8 form, which is recycled by deconjugation by ATG4(s). Conjugation of ATG8 with PE is enhanced by ATG12-ATG5 (red arrow). It is suggested that ATG8 (LC3 and GABARAP) serves various purposes during autophagy, including as elongating the membrane, recognizing cargo, closing the edge, moving autophagosomes, and connecting them to lysosomes (228).

Essential for autophagosome formation are core ATG genes (228). They are made up of 20 genes or gene families. These genes are responsible for producing six different proteins: ATG1 (known as ULK in mammals), ATG9 vesicle, ATG14-containing PtdIns 3-kinase complex, ATG2-ATG18 complex, ATG12 conjugation system, and ATG8 conjugation system make up the initial six complexes (107, 229). ATG8(s) and their counterparts in mammals are known as microtubule-associated protein 1 LC3 and GABARAP, respectively, unless the need arises to specifically name each ATG8. ATG8 and ATG12 systems serve a role similar to ubiquitin through covalent conjugation mechanisms (230–232). ATP-driven mechanism triggers the starting point of activating ATG12’s C-terminal glycine by E1-like enzyme ATG7 in ubiquitin-like protein. After that, thioester intermediates are gradually formed by ATG12, ATG7, and E2-like enzyme ATG10. The final stage involves the formation of an isopeptide bond between ATG12 and the lysine residue in ATG5. Two pairs of ATG12-ATG5 conjugates unite within a compound containing ATG16(L) dimer. The ATG8 system begins with the production of ATG8, a protein that is comparable to ubiquitin, in its proform state. Enzymes belonging to the ATG4 family cut the C-terminal region of this proform to expose a glycine residue. Afterwards, the ATG8 that has been processed is activated by ATG7 and ATG12 enzymes, with the help of E2-like enzyme ATG3, and then attaches to the PE head group. The membranes of autophagy contain ATG8-PE. Although ATG12 conjugation remains unchanged, ATG8-PE can be re-conjugated by ATG4. Remarkably, by interacting with ATG3, ATG12 enhances the ATG8-PE conjugation through ATG12-ATG5 conjugate functioning as an E3-like enzyme (233–236). ATG16L1’s membrane binding dictates where ATG8 lipidation occurs, despite not being essential for the process (237–239). ATG8 lipidation can occur on non-autophagy membranes, but it can be reversed through deconjugation carried out by ATG4, a process regulated by ATG1 and ULK1 An instance would be LC3-I representing modified ATG8, while LC3-II represents modified ATG8-PE. The articles that discussed the discovery of these two systems were part of the four “key publications” for Dr. Ohsumi’s 2016 Nobel Prize, as stated in references (230, 231). These publications significantly broadened the scope of research on autophagy. Even though ATG8 is the most commonly used marker for autophagosomes, ATG5 and ATG7 have been heavily utilized in knockout mouse research (**Figure 4**).

## 7 Non-autophagy functions of Beclin-1

Current research shows that Beclin-1 plays a role in processes beyond autophagy in addition to its known function as an autophagy effecter (240). Beclin-1 does not exhibit a pro-apoptotic function as it is solely a BH3 protein. It is interesting that Beclin-1’s control over autophagy and apoptosis is given through caspase-mediated cleavage (191). Caspases can cause Beclin-1 to be divided, resulting in cleaved N and C-terminal fragments that are no longer capable of inducing autophagy (241–243). Unlike the N-terminal Beclin-1 fragment containing the BH3 domain, only the C-terminal fragment is able to make cells more sensitive to signals that trigger apoptosis. Beclin-1-C must translocate to the mitochondria surface in order to induce the release of cytochrome c (241). Beclin-1-C enhances Bax translocation to mitochondria, leading to elevated apoptosis and reduced autophagy (244). Beclin-1 controls cell death and self-cannibalization, implying possible treatment benefits. It plays a role in multiple pathways of controlled cell death such as apoptosis, ferroptosis, and necroptosis. Certainly, new functions of Beclin-1 apart from autophagy have just become evident. Phosphorylation of Ser90, Ser93, and Ser96 in Beclin-1 by active AMPK leads to the formation of Beclin-1-SLC7A11 complex, which directly inhibits system Xc− activity and ultimately promotes ferroptosis (245). Another newly discovered, adversarial component of the necrosome complex is Beclin-1.Beclin-1 serves as a blocker of necroptosis by linking with phosphorylated MLKL in the necrosome complex, preventing MLKL from forming oligomers (246). Beclin-1 is involved in ferroptosis and necroptosis, and could be targeted for treatment in associated disorders.

In addition to autophagy, Beclin-1 has been linked to DNA damage responses that do not depend on autophagy, whether they need it or not. Activation of nonhomologous end joining (NHEJ) for DNA double-strand break repair necessitates a decrease in Beclin-1-UVRAG activity to uphold genome stability (247). Nuclear Beclin-1 and DNA topoisomerase IIβ are required for effective repair of DNA double-strand breaks caused by ionizing radiation in both autophagy-independent and autophagy-dependent DNA damage responses (248). Furthermore, Beclin-1 plays a role in tumor spreading, blood vessel formation, and immune system regulation through its actions that are not related to autophagy (249). In models of mouse melanoma tumors, animals with Beclin-1 (+/−) showed a more aggressive tumor growth and increased angiogenic activity, compared to mice with Beclin-1 wild-type, by boosting the protein expression and stability of hypoxia-inducible factor-2α (HIF-2α) (250). Increased levels of Beclin-1 lead to a decrease in angiogenesis in laboratory settings by lowering the production of matrix metalloprotease 9 and vascular endothelial growth factor (251). Beclin-1 also plays a role in regulating the immune system (252, 253). An example is how the lack of Beclin-1 is associated with the abnormal activation of the neutrophil MEKK3/p38 signaling pathway, increased B cell movement through the Cxcl9-Cxcr3 axis, and the conversion of precursor B cells into malignant ones (253).

## 8 Beclin-1 and ATG interaction in cancer

Beclin-1 and ATGs regulate autophagy and cancer progression. Understanding their interaction may lead to innovative cancer treatments. CUL3 E3 ligase promotes Beclin-1 degradation. This communication not just decreases autophagy function by blocking Beclin-1 but also includes KLHL38 as the adapter for substrates in the ubiquitination process controlled by the CUL3 E3 ligase complex. Moreover, elevated CUL3 levels are linked to a negative outlook in cases of breast and ovarian cancers, indicating its involvement in facilitating the growth of tumor cells in these scenarios (254). Additionally, Beclin-1 and ATGs may serve as predictive indicators in cancer. Out of 90 cancer cases, 55 showed positive Beclin-1 staining (61.1%) and 52 showed positive Atg5 staining (57.8%). Beclin-1 and Atg5 were both expressed in 40 tumors, but not in 23 tumors. Beclin-1 expression was associated with lymph node metastasis and tumor grade, whereas Atg5 expression was associated with tumor grade, clinical stage, tumor size, and lymph node metastasis. Beclin-1 patients showed increased 3-year overall survival (OS) rates and improved time to recurrence (TTR). However, there were no significant differences in survival between groups with positive and negative Atg5. Patients who displayed positive levels of both Beclin-1 and Atg5 had notably improved 3-year OS and TTR compared to those who had negative levels of both (p=0.022 and p=0.026, respectively) (255). Hence, ATGs and Beclin-1 serve as dependable prognostic indicators in cancer (256).

ATGs have the ability to also control Beclin-1 in a meaningful way. In Ca Ski cells, RCE-4 disrupted the formation of the Bcl-2-Beclin 1 complex through various pathways, with ATG 4B proteins playing an important role. It was shown that using an ATG 4B siRNA plasmid led to a significant increase in Ca Ski cell sensitivity by reducing ATG 4B expression, as proven by RCE-4 (257). The regulation of Beclin-1 and ATG2 is essential in the autophagy mechanisms related to cancer treatment. Exposure to cisplatin in A549 cells increased indicators of both autophagy and apoptosis, including conversion of LC3B-I/II, LC3B puncta, and formation of autophagosomes. Cisplatin also raised the mRNA and protein quantities of autophagy proteins Beclin-1 and Atg5. Nonetheless, it did not cause a substantial change in the levels of expression of serine/threonine-protein kinase ULK1, Atg3, Atg7, Atg12, and sequestosome-1. Additionally, silencing Atg5 and Beclin-1 with small interfering RNA (siRNA) led to decreased autophagy reactions to cisplatin, heightened caspase-3 cleavage, and diminished cell viability (89).

Although inhibiting apoptosis by silencing BAX and BAK, obatoclax still induced acute toxicity and loss of clonogenicity through activation of mitochondrial apoptosis via the BCL-2 antagonist killer/BAX pathway. Obatoclax greatly decreased cell viability while also keeping the integrity of the plasma membrane. This therapy also led to a decrease in 6 kinase S phosphorylation, ongoing LC3 autophagy processing, and significant vacuolation of the ultrastructure, which is unusual for autophagy. Unexpectedly, knocking down Beclin-1 did not affect the processing of LC3. Even though the suppression of autophagy-related protein 7 (Atg7) stopped LC3 processing, it did not impact the decline in clonogenicity or structural changes caused by obatoclax. Interestingly, inhibiting Atg7 with siRNA did not stop cell death in BAX/BAK mutant mouse embryonic fibroblasts caused by obatoclax. In line with apoptosis, cells that were not affected by obatoclax exhibited decreased LC3 processing and survival (258).

Blocking autophagy increases how well tumor cells react to cisplatin treatment. Cisplatin-sensitive FaDu cells have the potential to generate cystatin-resistant FaDu cells in a stable manner. In cisplatin-resistant FaDu cells, there is an increased level of expression of autophagy-related genes and proteins, such as Beclin-1, when compared to the control group. Treating the FaDu cells with an autophagy inhibitor after 24 hours led to a significant decrease in cell survival, higher rates of apoptosis, and notable impact on cell cycle, resulting in G1 phase arrest. There was a notable decrease in the quantity of autophagy vacuoles in the group treated with 3-MA. After being treated with 3-MA, p62 expression rose due to the interruption of autophagy flux. Furthermore, there was a notable decrease in the levels of Beclin-1, LC3-I, LC3-II, and Atg-5 proteins (259).

## 9 Beclin-1 and AMPK crosstalk

The relationship between Beclin-1 and AMPK is influenced by the curcumin analog ZYX01. ZYX01 triggers cell death in A549 cells through autophagy in a way that is influenced by both the dose and timing. ZYX01 treatment resulted in alterations in the LC3-II/LC3-I ratio, Beclin-1 expression, and p62 levels as indicated by Western blot analysis. Exposure to ZYX01 verified the activation of the AMPK/ULK1/Beclin-1 signaling pathway in A549 cells. ZYX01’s effect on A549 cell migration was evaluated through wound healing and transwell tests (260). Allyl Isothiocyanate (AITC) plays a role in controlling Beclin-1 in prostate cancer. AITC increases the amount of LC3-II protein in RV1 and PC3 cells over time, promoting autophagy. AITC does not impact PrEC. Blocking of autophagy in cells treated with AITC resulted in decreased viability and increased apoptosis, indicating a potential protective role of autophagy. Cells were exposed to varying AITC concentrations and autophagy marker levels were assessed at different time intervals. It was discovered that cells exposed to AITC showed activation of numerous pathways. Phosphorylated mTOR, ERK, AMPK, JNK, and p38 were specifically phosphorylated in the cells treated with AITC, with ERK, AMPK, and JNK being activated. AITC did not change the levels of Akt and its associated proteins. Nevertheless, despite prior treatment with targeted inhibitors like rapamycin, LY294002, and PI-103, autophagy induction persisted to rise as AITC triggered the ERK, AMPK, and JNK signaling pathways. AITC also induced an increase in the expression of Beclin-1. The study showed that using its specific siRNA to block Beclin-1 decreases the level of autophagy caused by AITC, suggesting that Beclin-1 is essential for AITC-induced autophagy (261). LETM1 controls both autophagy and apoptosis in liver cancer, with elevated levels found in hepatocellular carcinoma tissue, cell lines, and linked to a negative outlook. Depletion of LETM1 triggers apoptosis, autophagy, and hindrance of growth in liver cancer cell lines. AMPK phosphorylation of Bcl-2 after LETM1 depletion caused disruption in the Beclin-1/Bcl-2 complex (262).

## 10 Beclin-1 and mTOR interaction in autophagy regulation

In several experiments, the levels of expression for mTOR and Beclin-1 are evaluated separately as well as in relation to their interaction. Xie-Bai-San (XBS) was discovered to regulate both mTOR and Beclin-1. XBS inhibits gefitinib-induced autophagy in NSCLC cells, promoting cell death and hindering growth. It enhances p-mTOR and Bcl-2 levels while decreasing Beclin-1 levels, without affecting autophagosome-lysosome fusion or lysosome activity. XBS improves Bcl-2 and Beclin-1 communication, making it an effective treatment for gefitinib-resistant non-small cell lung cancer (NSCLC). Increased expression of Beclin-1 in NSCLC cells leads to enhanced cell growth and reduces cell death caused by XBS (263). Another element that controls autophagy in liver cancer is Oroxylin A. Oroxylin A was utilized to trigger Beclin 1-facilitated macroautophagy in the HepG2 cell line of human hepatocellular carcinoma. Within 12 hours, LC3-I in cells exposed to 80 μM oroxylin A changes to LC3-II and binds to the autophagosomal membrane, becoming water-insoluble. Nonetheless, the breakdown of autophagosomes by lysosomes/vacuoles is hindered after 24 hours as a result of cell death triggered by oroxylin A. Moreover, oroxylin A substantially inhibits the PI3K-PTEN-Akt-mTOR signaling pathway. Utilizing siBeclin 1 with 3-methyladenine (3-MA), which hinders autophagy, and enhancing autophagy-related genes like Atg5 and Atg7, can aid in establishing if autophagy granule protein (AGP) serves as a PCD mechanism (264).

LTX-315, a polypeptide, controls the Beclin-1/PI3K/mTOR axis to adjust autophagy and drug responsiveness in ovarian cancer. DDP-resistant ovarian cancer cell models were created, and LTX-315 treatment decreased the IC50 of DDP. The use of LTX-315 halted the advancement of cell cycle, raised apoptosis rates, and boosted levels of cleaved caspase-3, cleaved PARP, and Bax in ovarian cancer cells. Additionally, the use of LTX-315 decreased levels of Bcl-2 and impeded cellular movement and penetration. It also raised Beclin-1 levels and changed the levels of phosphorylated Akt (p-Akt) and phosphorylated mTOR (p-mTOR). However, 3-MA managed to partially mitigate the effects of LTX-315 on OC cells (265). It is understood that mTOR plays a role in controlling tumor growth and spread by regulating Beclin-1 (266).

The exact function of Interleukin-7 (IL-7) in controlling tumor cell autophagy, lymphangiogenesis, growth, and cell death is not completely clear; studies are centered on mTOR and Beclin-1. IL-7 was discovered to lower Beclin-1 levels and activate the PI3 K/Akt/mTOR pathway in lung cancer cells. Furthermore, the levels of Beclin-1 and mTOR are closely linked to the clinical stage and outcome of NSCLC patients. In cases of lung cancer, IL-7R, mTOR, and tumor stage are important predictors of prognosis (267).

Prodigiosin (PG), found in gram-negative bacteria, is a strong inducer of cell death and a red pigment that does not dissolve. It has shown effectiveness against lung cancer by inhibiting half of the maximum tumor growth at a dose of 10 μM in cells that are both sensitive and resistant to doxorubicin, suggesting comparable toxic effects. Autophagy was indicated in both cell types due to a slight rise in the sub-G_1_ phase and an increase in the levels of microtubule-associated proteins 1A/1B light chain 3B-phosphatidylethanolamine conjugate (LC3-II). Moreover, an increase in cleaved-poly ADP ribose polymerase (cleaved-PARP) suggested a subset susceptible to cell death. Impeding the PI3K-p85/Akt/mTOR signaling pathways hindered autophagy initiation in both cell types. Nonetheless, PG-induced autophagy was associated with the decrease of Beclin-1/PI3K-Class III, indicating the triggering of alternative autophagy mechanisms. While the growth of tumors in the animals’ tracheas increased during PG treatment, the therapy effectively decreased their size, validating PG’s effectiveness against both Dox-S and Dox-R lung cancers (92). Piceatannol’s inhibition of Beclin-1 may hinder the advancement of gastric cancer. Piceatannol boosts protein binding of UV radiation resistance-associated genes and supports Beclin-1-dependent autophagy signaling while also interfering with Beclin-1 and Bcl-2 interactions. Everolimus, a type of mTOR inhibitor, stimulates autophagy to enhance the anticancer effects of piceatannol (268).

## 11 Beclin-1-mediated autophagy regulation in cancer drug resistance and radioresistance

Chemotherapy and radiotherapy are the primary conventional treatments for cancer. These treatment methods are commonly used to control cell death and genetic damage in cancer cells hindering their advancement. Nonetheless, if the tumor cells spread and migrate to different parts of the body, it becomes challenging to completely eliminate all of them, leading to potential tumor relapse and recurrence. Yet, a major obstacle in cancer treatment currently lies in tumor cells’ capacity to become resistant to cancer medications. The resistance frequently occurs due to the dysfunction of molecular pathways and drug efflux transporters, among other reasons, leading to a difficult challenge for researchers (269, 270). The current portion concentrates on Beclin-1-mediated autophagy regulation’s involvement in cancer drug resistance and radioresistance. Hypoxia in glioblastoma (GBM) can lead to drug resistance. It has been shown that HIF1A-triggered autophagy plays a crucial role in the radioresistance of GBM. Suppressing HIF1A increased the radiosensitivity of GBM in both lab settings and living organisms. One possible explanation for this impact is the decrease in Beclin-1 caused by the suppression of HIF1A, which could provide protection for cells. Another option is that the blocking effect of 3-MA is partly influenced by Beclin-1; therefore, boosting Beclin-1 levels could offset the sensitivity caused by 3-MA. Moreover, there could be undiscovered resistance pathways that help cells sustain their radioresistance. Hence, the autophagy controlled by HIF1A and Beclin-1 is essential in the radioresistance seen in GBM (271). miR-216a has also been proven to control Beclin-1, impacting radiosensitivity. MiR-216a levels were noticeably decreased compared to controls, while autophagy activity was elevated. The presence of miR-216a inhibited autophagy and the key autophagy factor Beclin-1 by directly interacting with the 3’-UTR of Beclin-1. Moreover, increased levels of miR-216a in radio-resistant pancreatic cancer cells resulted in improved apoptosis under irradiated conditions, along with reduced cell proliferation and colony formation abilities. Increasing the levels of Beclin-1 reversed the impacts of reducing miR-216a. In general, miR-216a increases xenograft tumors’ responsiveness to radiotherapy and prevents radiation-triggered autophagy by controlling Beclin-1 (272).

Nevertheless, the majority of research has concentrated on the role of Beclin-1-mediated autophagy in chemoresistance. EGFR has the ability to control Beclin-1 in order to inhibit autophagy and manage chemoresistance. When there is an active EGFR signaling pathway, Beclin-1 may be contained, resulting in its tyrosine phosphorylation, increased inhibitory binding, and the interference of its linked VPS34 kinase function. In NSCLC cells with sensitive EGFR mutations, EGFR kinase inhibitors can trigger autophagy by disrupting tyrosine phosphorylation of Beclin-1, leading to enhanced tumor growth and resistance to TKI treatment (273).

Interplay between non-coding RNAs can influence drug sensitivity by regulating Beclin-1. Increased levels of PVT1 were linked to unfavorable outcomes in NSCLC patients (*P < 0.05). Overexpressing PVT1 negatively affected A549 cells, while reducing PVT1 intensified cisplatin’s effects on cell viability and apoptosis in A549/DDP cells. PVT1 upregulation promoted autophagy and tumor growth in NSCLC cells, with miR-216b interacting with PVT1 and Beclin-1. Beclin-1 reversed miR-216b’s impact on autophagy and apoptosis, and PVT1 and miR-216b communication controlled Beclin-1 expression. (*P < 0.05, #P < 0.05) (274).

In human cancers, increasing drug sensitivity is possible by inhibiting protective autophagy. In particular, miR-30a hinders Beclin-1, decreasing autophagy and enhancing the susceptibility of gastrointestinal tumors to imatinib (275). Low miR-30a linked to osteosarcoma drug resistance, upregulating Beclin-1 for increased survival autophagy (276). Research showed that reducing miR-30a-5p levels in lung cancer can increase Beclin-1 expression, causing drug resistance. Similar effects were seen in colon cancer (277). Enhancing sensitivity to oxaliplatin in colon cancer can be achieved by suppressing the Beclin-1/autophagy axis through the restoration of miR-409-3p expression (278).

## 12 Beclin-1-mediated autophagy regulation and apoptosis

In apoptosis, proteins like caspases 3, 6, and 7 destroy cells by cutting numerous proteins (279, 280). The development of pores in target cells by perforin can initiate apoptosis, along with the release of granzymes from cytotoxic granules of T-cells and Natural Killer (NK) cells (281). DNA in the cell nucleus is usually packed in a dormant state due to the fact that the vast length of DNA would not be able to fit inside the cell if it was completely stretched out. This method consists of coiling DNA in a counter-clockwise direction around protein clusters to create nucleosomes. Nonetheless, somatic topoisomerase 2 enzyme has the ability to interfere with this arrangement by turning the DNA coils in a different direction. This process causes the DNA to resemble a ladder pattern when viewed on an agarose gel (282, 283). This occurrence does not only happen in nuclear apoptosis but also when cells are preparing and experiencing apoptosis. In this procedure, the nuclear membrane forms blebs, necessitating the breaking down of lamins by caspase 6. Moreover, a significant indicator of apoptosis, especially the activation of caspase 3, is the cleavage of PARP, which takes place during cell death (284). Serum M30, a form of cleaved cytokeratin 18 by caspase 3, is detected in patients with tumor response to treatment, signifying cell death (285, 286). Western blotting and the TUNEL assay are frequently utilized methods to identify cleaved caspases and fragmented DNA in tissue samples, showing presence of Serum M30, a caspase 3-cleaved cytokeratin 18, in patients with successful tumor responses. Immunohistochemistry detects granzyme B in cytolytic T cells inducing cell death (287). Flow cytometry is able to detect apoptosis in cultured cells by assessing different indicators like cleaved caspases (288), phosphatidylserine externalization, or sub-G1 DNA content. Furthermore, caspase enzyme functioning and assessments done on cells, along with imaging tests on humans, can also identify caspase activation (289, 290). The identification of apoptosis has made preclinical studies in drug development easier, utilizing PET scans to show markers like Granzyme B (291) as signals of drug effectiveness and impact in clinical specimens. Using standardized terminology for cell death enables the categorization of different forms of cell death and the use of suitable techniques for identifying these mechanisms (5).

Beclin-1 plays a crucial role in linking autophagy and apoptosis in the control of cancer advancement. It acts as a central regulator connecting these pathways. The pairing of bortezomib and mitomycin C mainly led to cell death and cytotoxicity, with little involvement of autophagy, either through additive or synergistic effects. This specific therapy resulted in Akt being inactivated, leading to the dephosphorylation of Beclin-1 at Ser 234/295. Dephosphorylating Beclin-1 inhibited autophagy and caused a pro-apoptotic effect, leading to the cleavage of Beclin-1 and disruption of the R-BiP/Beclin-1/p62 complex. Significantly, this combination greatly diminished autophagy, inactivated Akt, and triggered Beclin-1 cleavage *in vivo*, leading to a substantial decrease in the growth of intraperitoneal xenografted LS174T tumors in mice. Additionally, in both laboratory and live settings, LS174T xenografted tumors containing a mutated caspase 8 cleavage site of Beclin-1 showed significant resistance to the anti-tumor effects of the combined treatment (292).

Apoptosis caused by radiotherapy (RT) is affected by autophagy mediated by Beclin-1. Both apoptosis and autophagy are mechanisms that RT activates to cause death in thyroid cancer cells. Beclin-1 and LC3, proteins related to autophagy, saw an increase after exposure to radiation. Furthermore, blocking autophagy with 3MA and Beclin-1 siRNA increased radiation-induced cell death and led to higher levels of p53 expression (293).

The relationship between apoptosis and autophagy is significant in prostate tumors, with autophagy-like changes seen in miR-139-treated cancer cells. Although miR-139 antagomir successfully blocked the conversion of LC3-I to LC3-II through autophagy, miR-139 promoted this process. Confocal microscopy data additionally validated the elevated levels of LC3-II. MiR-139 controlled the regulation of two important molecules, mTOR and Beclin-1, in autophagy. After miR-139 treatment, there was an increase in the accumulation of cargo receptor protein p62 which is typically degraded during autophagy (294).

The primary and most effective component in the water extract of Scutellaria baicalensis Georgi’s root, known as Baicalein (BA), can effectively overcome cephalotin resistance. This discovery establishes a theoretical foundation for utilizing BA in clinical settings to combat cephalosporin-resistant strains and endorses its capacity as a promising alternative treatment option. BA was discovered to block cell growth and induce cell death in ovarian cancer cells. BA therapy led to elevated levels of vacuoles stained with acridine orange, puncta of GFP-LC3, and expression of LC3-II. Additionally, both the HEY and A2780 ovarian cancer cell lines showed increased cleavage of poly (ADP-ribose) polymerase (PARP) and decreased cell viability when treated with both chloroquine and BA. This implies that BA may induce cyto-protective autophagy in these cells. Reducing Beclin-1 rates in HT29 cells resulted in a decline in BA-induced LC3-II lipidation. BA treatment led to elevated rates of AKT phosphorylation at Ser473 and extracellular signal-regulated kinase (ERK) phosphorylation at Thr202/Thr204 (295), illustrating an association among autophagy, apoptosis, and the role of Beclin-1 (93, 242, 296–300).

## 13 Beclin-1-mediated autophagy regulation and ferroptosis

Lipid peroxidation, which is influenced by the amount of iron in cells, is the primary cause of ferroptosis. Regulation of ferroptosis is closely associated with different aspects of iron metabolism, such as iron absorption, transfer, retention, and use. The activation of lipid metabolic pathway enzymes, including LACS4, LPLAT5, LOX, and NOX, also impacts this process by promoting ferroptosis and lipid peroxidation. Furthermore, the cystine-glutamate antiporter (system xc−) is crucial in the classic pathway that prevents ferroptosis by aiding in the synthesis of glutathione (GSH), a necessary cofactor for glutathione peroxidase 4 (GPX4) to convert phospholipid hydroperoxides into alcohols. The system involving ferroptosis suppressor protein 1 (FSP1) and coenzyme Q10 (CoQ10) also plays a role in controlling phospholipid peroxidation. Iron metabolism affects ferroptosis by two main routes of non-heme iron intake in cells: uptake of TF-bound iron through TFR1 and uptake of non-TF-bound iron through ZIP14. The Fenton reaction, triggered by haem degradation and ferritinophagy mediated by nuclear receptor coactivator 4 (NCOA4), can result in heightened vulnerability to ferroptosis (301–308).

Beclin-1, an important controller of autophagy, plays a role in connections with ferroptosis and its related pathways, molecules, and proteins (309). Proteins that control various cellular functions, such as autophagy and cell death, have been identified through proteomic studies of the Beclin-1 interactome (191). Erastin triggers the formation of Beclin-1-SLC7A11 complexes, leading to ferroptosis activation. These complexes are said to not be necessary for cell death and do not contribute to ferroptosis triggered by type 2 activators (RSL3 and FIN56) (310). Moreover, by phosphorylating Beclin-1 at Ser90/93/96, PRKAA/AMPKα boosts the assembly of the Beclin-1-SLC7A11 complex, inhibits system xc−, and stimulates the resulting ferroptotic demise of cancer cells (311). Moreover, by phosphorylating Beclin-1 at Ser90/93/96, PRKAA/AMPKα boosts the assembly of the Beclin-1-SLC7A11 complex, inhibits system xc−, and stimulates the resulting ferroptotic demise of cancer cells (311). The Tat-Beclin-1 peptide boosts the erastin reaction in a laboratory setting or within a living organism (311). ELAVL1, present in normal livers, also produces Beclin-1 in stellate cells leading to an elevated chance of ferroptosis (312). This information backs the claim that Beclin-1 directly inhibits system xc− to facilitate the beginning of ferroptosis. Still unknown whether OTUB1 affects the stability of SLC7A11 by influencing the interaction between Beclin-1 and SLC7A11 (313).

Nanoparticles have been designed to regulate this process since the finding of autophagy-induced ferroptosis in treating cancer. Ultrasmall iron oxide nanostructures have been created with excellent water solubility to induce ferroptosis in glioblastoma. These nanoparticles also decrease the levels of factors that protect against ferroptosis.

Suppression of autophagy reduces the ability of these nanoparticles to induce ferroptosis, indicating a potential interaction between autophagy and ferroptosis. Noticeably, tiny iron oxide structures enhance Beclin-1 production, leading to increased ATG5 levels, promoting autophagy-triggered ferroptosis in the treatment of glioblastoma (**Table 3**) (310).

**Table 3:** Beclin-1-mediated autophagy regulation in cancer.

Molecular aspects	Outcome	Ref
CircHIPK3	CircHIPK3 disrupts the VCP and Beclin-1 complex to suppress autophagy in bladder tumor	(314)
Beclin-1	Piceatannol enhances the expression of Beclin-1-related autophagy to impair cancer malignancy and enhance the potential of everolimus in gastric tumor	(268)
miR-409-3p	miR-409-3p downregulates Beclin-1 to suppress autophagy and increase oxaliplatin sensitivity	(278)
Beclin-1	Allyl Isothiocyanate stimulates autophagy through Beclin-1 upregulation in prostate tumor	(261)
Beclin-1	Thymoquinone disrupts autophagy, LC3 and Beclin-1 in the treatment of breast cancer through suppressing growth and metastasis	(315)
Beclin-1 and ERK	Hydroxysafflor yellow A stimulates autophagy through Beclin-1 upregulation and ERK downregulation	(316)
Beclin-1	Aspirin induces triggers autophagy in hepatocellular carcinoma through Beclin-1 upregulation	(317)
miR-129-5p	Norcantharidin downregulates miR-129-5p to upregulate Beclin-1 in the induction of autophagy in prostate tumor	(84)
Beclin-1 CUL3	Beclin-1 degradation by CUL3 impairs autophagy	(254)
USP5-Beclin 1	Silencing USP5-Beclin 1 axis disrupts autophagy and promotes senescence	(318)
XIAP and cIAP1	Overexpression of XIAP and cIAP1 can mediate Beclin-1-related autophagy through increasing NFκB expression	(297)

## 14 Beclin-1 modulators in cancer therapy

**Figure 5:**
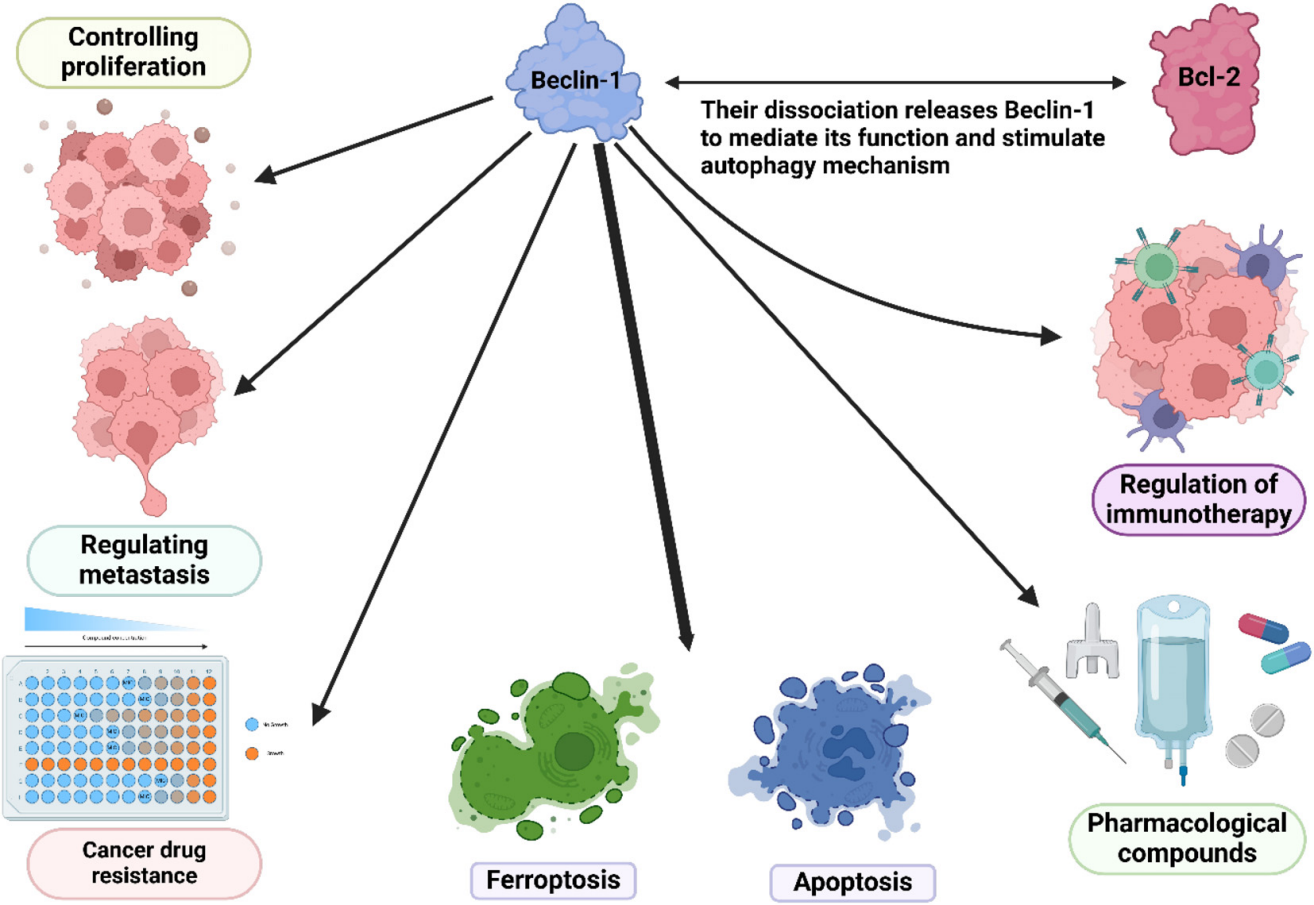
The overall function of Beclin-1 in the regulation of cancer hallmarks and cell death mechanisms.

Quercetin suppressed HeLa cell proliferation and triggered autophagy in a manner that was dependent on the dosage used (319). It decreased cell growth, elevated Beclin-1 and LC3-I/II levels, and lowered S6K1 phosphorylation levels (320). Examination of omics data in LGG discovered that ZFP36L2 and RAB13 are involved in regulating autophagy by enhancing Beclin-1 and other crucial autophagy elements. The presence of Gallic acid, which can block RAB13, has been shown to reduce autophagy and induce cell death in SW1088 cells (62). The collaboration of nobiletin and vorinostat in small cell lung cancer (SCLC) causes autophagy and apoptosis by interfering with the BCL-2 and Beclin-1 complex, releasing Beclin-1 to initiate autophagy and inhibiting PI3K-AKT-mTOR signaling, suggesting nobiletin’s potential as a BH3 mimetic for SCLC combination treatment (321). Resveratrol increases Beclin-1, LC3-II, and p53 expression in A549 NSCLC cells, inducing apoptosis and autophagy, reducing cell survival via p53 pathway activation. Autophagy inhibition partly reverses this effect (322). Resveratrol alleviates doxorubicin-induced heart damage by inhibiting S6K1 and reducing autophagy. Beclin-1 overexpression counteracts Resveratrol’s protective effects (323). Resveratrol induces apoptosis in MCF-7 breast cancer cells through caspase-dependent and -independent pathways. This unconventional type of autophagy cell death is significant in caspase-3-deficient cells (324). Resveratrol induces cell death in MCF-7 breast cancer cells through caspase-dependent and caspase-independent pathways, triggering macroautophagy independently of Beclin 1. This atypical form of autophagic cell death is especially important in cells that do not have caspase-3 (325). Resveratrol induces a defensive autophagy mechanism that boosts cancer cell demise by raising caspase activation and cell death in the absence of autophagy; this mechanism includes Beclin-1 binding with p53 and results in impaired mitochondrial function marked by decreased mtDNA content (326). Resveratrol enhances cell death in ovarian cancer cells by blocking STAT3 signaling, leading to higher autophagosome production and elimination of mitochondria, ultimately leading to inhibition of growth and cell death (327). Resveratrol prevents the spread of breast cancer and its growth in other areas by reversing TGF-β1-induced EMT and boosting autophagy through the SIRT3/AMPK pathway (328). Therefore, increasing research indicates the significance of pharmacological compounds in modulating Beclin-1 in human cancer and overseeing autophagy (153, 260, 329–332). **Figure 5** provides a comprehensive view of Beclin-1’s regulation of autophagy in human cancer.

## 15 The multifaceted role of Beclin-1 in tumor immunology and drug sensitivity

Beclin-1 is a key regulatory factor in the autophagy process, and research advancements regarding its role in the tumor immune microenvironment, immunotherapy, and drug sensitivity indicate its potential application value in cancer treatment (333, 334). In the tumor immune microenvironment, Beclin-1 influences the interaction between tumor cells and immune cells by regulating autophagy. Specifically, the expression of Beclin-1 can alter the polarization state of tumor-associated macrophages (TAMs), enhancing their immunosuppressive function and thus promoting tumor development (252, 335). Additionally, Beclin-1 affects the antigen presentation capability of tumor cells, altering how tumor cells are recognized and attacked by the immune system, which allows tumor cells to partially evade immune surveillance. Regarding immunotherapy, studies have found that Beclin-1 may enhance the efficacy of immune checkpoint inhibitors, making it a potential target for combination therapy. By combining Beclin-1 modulators with immunotherapeutic agents, it is possible to significantly improve anti-tumor immune responses (336). Furthermore, the expression level of Beclin-1 may serve as a biomarker for evaluating patient responses to immunotherapy, aiding clinicians in developing more targeted personalized treatment plans (337). In terms of drug sensitivity, Beclin-1 also plays an important role in the sensitivity of tumor cells to chemotherapy. Research indicates that the regulation of autophagy is closely linked to the survival and death of tumor cells, with the overexpression of Beclin-1 being closely associated with chemotherapy resistance (336, 338). Therefore, based on the function of Beclin-1, researchers are exploring drugs that target autophagy regulation to enhance the sensitivity of tumor cells to chemotherapy and targeted therapy. Overall, the complex roles of Beclin-1 in the tumor immune microenvironment, immunotherapy, and drug sensitivity make it an important focus of cancer treatment research. Future studies need to further investigate the specific mechanisms of Beclin-1 to develop more effective therapeutic strategies, particularly in personalized and combination therapies, which will help improve the survival rates and quality of life for cancer patients.

## 16 Conclusion

Autophagy is one of the most frequently disrupted processes in cancer. Autophagy dysregulation has been noted in both solid and hematological cancers. Additionally, autophagy is a versatile process that controls different characteristics of cancer. Autophagy in clinical trials led to new cancer drugs. It plays roles in both cell survival and death. Hence, dual blocking and stimulation of autophagy are significant. Even though several autophagy regulators like mTOR, ULK1, and AMPK have been identified, Beclin-1 is considered a crucial controller of autophagy in cancer. The present study shows that controlling autophagy through Beclin-1 can impact cell growth, resistance to drugs, and response to radiotherapy. In addition, Beclin-1 regulates autophagy in human cancers by interacting with ATGs, mTOR, and AMPK. Hence, manipulating Beclin-1 can impact regulators both before and after it in the signaling pathway. Additionally, there have been reports stating that Beclin-1 controls autophagy, which can potentially affect apoptosis and ferroptosis in human cancer. Hence, purposeful adjustment of Beclin-1 and autophagy could greatly increase cell mortality in human cancers. This evaluation can be viewed from various angles. In terms of fundamental research, the dysregulation of Beclin-1 has been extensively documented in numerous studies. Nevertheless, Beclin-1 poses difficulties because of its dual function. While its role in cancer is intricate, Beclin-1 is crucial clinically, as its levels can impact how cancer patients respond to chemotherapy. Furthermore, Beclin-1 has the potential to influence survival, prognosis, growth, and tumor recurrence. Hence, regulating the Beclin-1 expression can result in the creation of innovative treatments. Beclin-1 plays a crucial role in regulating autophagy, impacting immunogenic cell death, necroptosis, and ferroptosis. This is important for timely detection and outcome prediction in cancer patients, with potential implications for cancer treatment (21, 339–343). Hence, it is recommended that upcoming research assess the relationship between the Beclin-1/autophagy axis and immunogenic cell death in treating human cancers. Moreover, addressing cancer is greatly challenged by drug resistance and radioresistance (344–347). Thus, the utilization of immunotherapy and checkpoint inhibitors has risen for the removal of tumors (348–352). Current research studies are limited because they fail to consider the impact of the Beclin-1/autophagy axis on immune interactions and how it affects cancer cells’ reactions to immune checkpoint inhibitors. Autophagy offers significant clinical insights in addition to Beclin-1’s basic and clinical relevance. Autophagy genes as well as non-coding RNAs play a crucial role in evaluating cancer diagnosis and treatment.

Beclin-1 is essential for regulating various cellular processes like autophagy, apoptosis, and ferroptosis. Beclin-1, an essential initiator of autophagy, interacts with VPS34, a type III phosphatidylinositol 3-kinase, to start the process of autophagy vesicle formation. A chemical creates PI3P, drawing in autophagy proteins to create phagophores, which then transform into autophagosomes. Beclin-1 aids in autophagosome development and breakdown, merging them with lysosomes for material degradation. Beclin-1 interactions with ATG proteins and LC3 enhance autophagy, emphasizing its role in cellular balance. When it comes to apoptosis, Beclin-1 shows a complicated connection and can often function as a pro-apoptotic factor in specific situations. Beclin-1 impacts programmed cell death by interacting with caspases, like regulating caspase-3, crucial for cell death. It binds to Bcl-2 proteins, trapping anti-apoptotic ones and inducing apoptosis. Also, it affects mitochondria function, releasing pro-apoptotic factors, triggering apoptosis. Beclin-1 can determine cell fate through supporting survival via autophagy or aiding apoptosis under stress. It also regulates ferroptosis, dependent on iron and lipid peroxides. Autophagy breaks down damaged lipids, preventing lipid peroxidation and reducing ferroptotic cell death. Furthermore, Beclin-1 can help regulate the levels of reactive oxygen species (ROS) by promoting the breakdown of impaired organelles, consequently reducing the oxidative stress linked to ferroptosis. Beclin-1’s interactions with GPX4 influence cell susceptibility to ferroptosis. Beclin-1, a flexible protein, controls autophagy, apoptosis, and ferroptosis processes, making it a possible treatment focus in illnesses. Utilizing Beclin-1 in treatments shows promise but requires caution due to potential challenges. Beclin-1 regulates autophagy and is vital for cellular balance, stress response, and cell death. Enhancing autophagy by improving Beclin-1 function is being studied to overcome blockages. Therapies targeting Beclin-1 levels can aid in removing harmful substances and supporting cell survival by restoring autophagy. However, Beclin-1’s involvement in autophagy and apoptosis complicates its therapeutic use. While increasing autophagy with Beclin-1 can help in some cases, it might promote cancer growth by supporting cell survival. Changing Beclin-1 levels could disrupt the balance between autophagy and apoptosis, leading to issues like increased cell death or reduced response to chemotherapy. Using Beclin-1 modulators may also trigger inflammatory responses, complicating treatment. Disruption of Beclin-1 can alter cytokine production and immune cell function, impacting inflammatory conditions and infection defense. Understanding Beclin-1’s roles and potential side effects is crucial for developing treatments across diseases. Future research should identify patient groups benefiting from Beclin-1 adjustments and develop targeted delivery methods to enhance treatment efficacy and minimize side effects. Further investigation is essential to understand Beclin-1’s intricate role in cancer by studying its interactions with pathways such as autophagy. Autophagy is interconnected with signaling pathways controlling cell survival, growth, and death, like the PI3K pathway crucial for autophagy initiation. Disrupted PI3K pathway functioning can impact autophagy levels, influencing cancer development and treatment response. Beclin-1’s interaction with the Bcl-2 protein family highlights the balance between cell survival and death. Understanding how Beclin-1 integrates signals from these pathways can uncover how cancer cells react to stress and treatment evasion. Cancer cells change metabolism to survive, possibly through Beclin-1 affecting metabolic reprogramming by regulating autophagy, which links to glucose and oxidative stress. Research must also focus on autophagy’s connection with immune signaling pathways, impacting tumor immunogenicity and response to immunotherapies. Fully grasping Beclin-1’s involvement in cancer requires exploring its links to cellular pathways for new treatment strategies. Future studies should elucidate these relationships for advancements in cancer treatment and patient outcomes.
